# Migratory Dermal Dendritic Cells Act as Rapid Sensors of Protozoan Parasites

**DOI:** 10.1371/journal.ppat.1000222

**Published:** 2008-11-28

**Authors:** Lai Guan Ng, Alice Hsu, Michael A. Mandell, Ben Roediger, Christoph Hoeller, Paulus Mrass, Amaya Iparraguirre, Lois L. Cavanagh, James A. Triccas, Stephen M. Beverley, Phillip Scott, Wolfgang Weninger

**Affiliations:** 1 The Wistar Institute, Philadelphia, Pennsylvania, United States of America; 2 The Centenary Institute for Cancer Medicine and Cell Biology, Newtown, New South Wales, Australia; 3 Department of Pathobiology, School of Veterinary Medicine, University of Pennsylvania, Philadelphia, Pennsylvania, United States of America; 4 Department of Molecular Microbiology, Washington University School of Medicine, St. Louis, Missouri, United States of America; 5 Department of Dermatology, Medical University of Vienna, Vienna, Austria; 6 Microbial Pathogenesis and Immunity Group, Discipline of Infectious Diseases and Immunology, University of Sydney, Camperdown, New South Wales, Australia; 7 Discipline of Dermatology, University of Sydney, Camperdown, New South Wales, Australia; Queensland Institute of Medical Research, Australia

## Abstract

Dendritic cells (DC), including those of the skin, act as sentinels for intruding microorganisms. In the epidermis, DC (termed Langerhans cells, LC) are sessile and screen their microenvironment through occasional movements of their dendrites. The spatio-temporal orchestration of antigen encounter by dermal DC (DDC) is not known. Since these cells are thought to be instrumental in the initiation of immune responses during infection, we investigated their behavior directly within their natural microenvironment using intravital two-photon microscopy. Surprisingly, we found that, under homeostatic conditions, DDC were highly motile, continuously crawling through the interstitial space in a Gα_i_ protein-coupled receptor–dependent manner. However, within minutes after intradermal delivery of the protozoan parasite *Leishmania major*, DDC became immobile and incorporated multiple parasites into cytosolic vacuoles. Parasite uptake occurred through the extension of long, highly dynamic pseudopods capable of tracking and engulfing parasites. This was then followed by rapid dendrite retraction towards the cell body. DDC were proficient at discriminating between parasites and inert particles, and parasite uptake was independent of the presence of neutrophils. Together, our study has visualized the dynamics and microenvironmental context of parasite encounter by an innate immune cell subset during the initiation of the immune response. Our results uncover a unique migratory tissue surveillance program of DDC that ensures the rapid detection of pathogens.

## Author Summary

Cutaneous Leishmaniasis is a difficult-to-treat disease affecting millions of people worldwide. Hence, there is high demand for the development of vaccines against *Leishmania* parasites, begging for a better understanding of immune responses against this pathogen. Dendritic cells, as part of the innate immune system, are thought to act as gatekeepers against intruding pathogens. However, their behavior in the context of intact tissues is incompletely understood. Here, we have used intravital two-photon microscopy to visualize the behavior of skin resident dendritic cells in real time, both in the steady-state and upon parasite encounter. We have found that migratory dermal dendritic cells are capable of rapidly sensing *Leishmania* parasites injected into the skin. This occurred through the formation of highly motile cellular processes capable of engulfing parasites, followed by parasite uptake into the cell. Together, our study provides a new vista of the orchestration of host cell–pathogen encounter in the three-dimensional context of intact tissues. Our results serve as the basis for a better understanding of the dynamic regulation of tissue surveillance by dendritic cells.

## Introduction

The skin is the interface between the environment and internal tissues. Dendritic cells (DC), as part of the body's innate immune defense, are strategically positioned in this organ; the epidermis is the home of Langerhans cells (LC), while the dermis harbors dermal DC (DDC). The main function of DC is believed to be the recognition and processing of foreign antigens, and subsequent presentation to naïve T cells [1]. DC normally reside in an immature state in the skin. Upon antigen encounter in the presence of “danger signals”, such as proinflammatory cytokines, DC undergo maturation enabling their migration to draining lymph nodes (LN) [2]. Accumulating evidence suggests that DDC may be responsible for the transport of pathogens to draining LN [3]–[6]; in certain infections, for example with herpes simplex virus, DDC act as an antigen shuttle, i.e. they transfer antigen to LN-resident CD8^+^ DC, which subsequently present it to T cells [7]. In other infections, including those with *Leishmania* parasites, they may present antigen directly to T cells [6].

Using intravital confocal microscopy, LC in the skin were found to be immobile with occasional repetitive dendrite movement, termed dendrite surveillance extension and retraction cycling habitude (dSEARCH) [4],[8]. In contrast to LC, very little is known about the migratory and interactive behavior of DDC. This is of significance, as during certain infections DDC may come into close contact with microorganisms, and it is unclear whether DDC are capable of detecting living pathogens directly or take up antigens from dying infected cells or dead pathogens. Since these initial events of an immune response are likely to determine the magnitude and quality of T cell and B cell immunity, it is important to decipher the events of pathogen encounter directly *in situ*.

Cutaneous Leishmaniasis is a disease caused by a large group of protozoan parasites belonging to the Genus *Leishmania*, including *L. major*. It serves as a paradigmatic skin infection, as promastigote stage parasites are directly deposited into the dermis during sand fly bites [9]. While it is thought that the parasites then infect innate immune cells in the skin, primarily macrophages [10],[11], the precise events occurring at the time of infection are not well defined. After entering cells, the parasites rapidly transform to the amastigote form, a rounded non-flagellated stage, which survives and multiplies within the phagolysosome (parasitophorous vacuole, PV) up until the time of cell rupture. After several weeks a lesion at the site of infection develops that is primarily composed of infected and inflammatory cells [12],[13]. In some cases, these lesions are able to resolve over several months, while in others the lesions are chronic and can be associated with severe disease [9]. Current treatment options are scarce, therefore begging for the development of prophylactic vaccines. A prerequisite for this is a thorough understanding of the immune response against the parasites.

Several studies have investigated the response of cutaneous DC to *Leishmania spp*. Initial reports suggested that LC are infectable by *Leishmania*, migrate to LN and activate CD4^+^ T cells [14]. However, more recently these findings have been questioned as DC harboring parasites in LN do not express the LC marker langerin [6]. Also, mice that lack MHC class II expression in LC but not DDC resolve infection similarly to wildtype animals [15]. While several investigators have suggested that DDC transport *Leishmania* to the paracortex of draining LN [5],[6], others have questioned the role of cutaneous DC during early infection altogether [16],[17]. At later stages of disease, monocyte-derived DC may differentiate directly within the inflamed skin, and then migrate to draining LN where they induce CD4^+^ T cell activation [18]. To gain further insight into this controversy, i.e. what is the nature of parasite-DC encounter during early infection, ideally, *Leishmania* infections should be visualized directly in the natural microenvironment of the skin.

In the present study, we made use of intravital two-photon microscopy (2P-IVM) to address the following questions: 1. What is the steady-state behavior of DDC? 2. How do DDC respond to danger signals? And 3. Do cutaneous DC take up *Leishmania* parasites in the early phase of infection, and if so, what are the dynamics of this process? Surprisingly, we found that DDC were migratory under homeostatic conditions, which is in stark contrast to their epithelial counterparts. After encountering danger signals, DDC underwent a morphological transition into immobile, dendritic-shaped cells. At this point, the cells were capable of taking up parasites through the elaboration of motile pseudopods. Together, these results shed new light on the dynamics and anatomy of host-pathogen interactions.

## Results

### CD11c-YFP Mice Enable Visualization of Cutaneous DC

**Figure 1:**
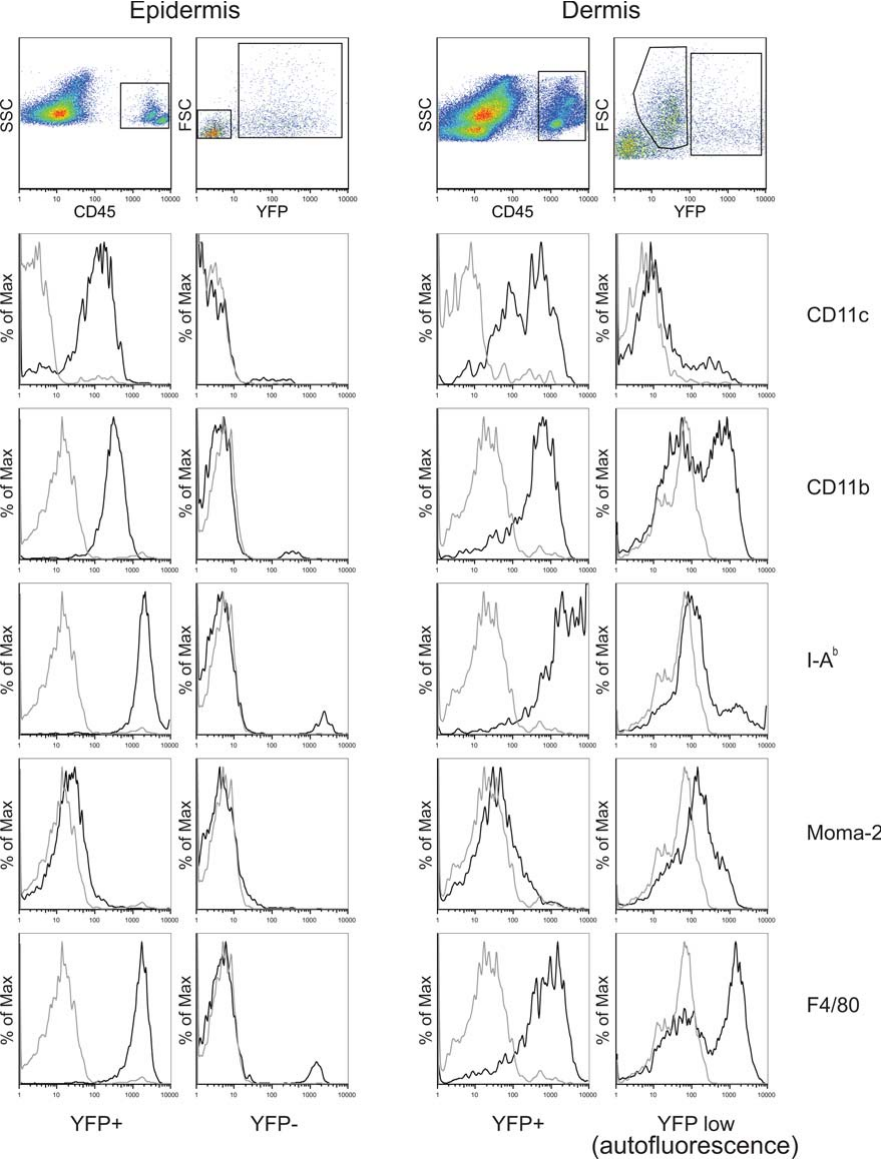
Phenotypic characterization of CD11c-YFP^+^ cells in ear skin. Flow cytometric analyses of surface markers expressed by epidermal and dermal cells from CD11c-YFP mice. The histogram plots were pre-gated on forward (FSC) and side-scatter (SSC) profiles. SSC/CD45 and FSC/YFP plots are shown for clear distinction of individual cell populations. Representative plots from 3 to 4 animals are shown.

In order to visualize the behavior of LC and DDC, we made use of CD11c-YFP mice [19], in which DC express high levels of cytoplasmic YFP. To ascertain that skin DC expressed YFP, we analyzed single cell suspensions prepared from separated epidermis and dermis by flow cytometry (Figure 1). CD45^+^YFP^+^ epidermal cells were CD11c^+^CD11b^+^F4/80^+^I-A^b+^ (Figure 1), and immunofluorescence staining of tissue sections showed that langerin expressing YFP^+^ cells displayed the characteristic morphology of LC (data not shown). In the dermis, CD45^+^YFP^+^ cells were CD11c^+^CD11b^+^F4/80^+^I-A^b-high^, and therefore represented DDC [20]. We also detected a subset of CD45^+^YFP^low^ cells within the dermis. However, this signal was due to autofluorescence, rather than specific YFP expression, as a similar population of cells was also found in wildtype animals (Figure S1A). These cells were CD11c^−^CD11b^+^F4/80^+^Moma-2^+^I-A^b-low^ thereby resembling dermal macrophages [20]. The fluorescence intensity of these cells was, on average, 50 times dimmer than the YFP signal from DDC. Since, under our 2P imaging conditions, we did not detect any signal in the dermis of wildtype animals (Figure S1B), we concluded that LC and DDC in CD11c-YFP mice can be detected by means of specific YFP expression, while other hematopoietic cell subsets remain undetectable.

**Figure 2:**
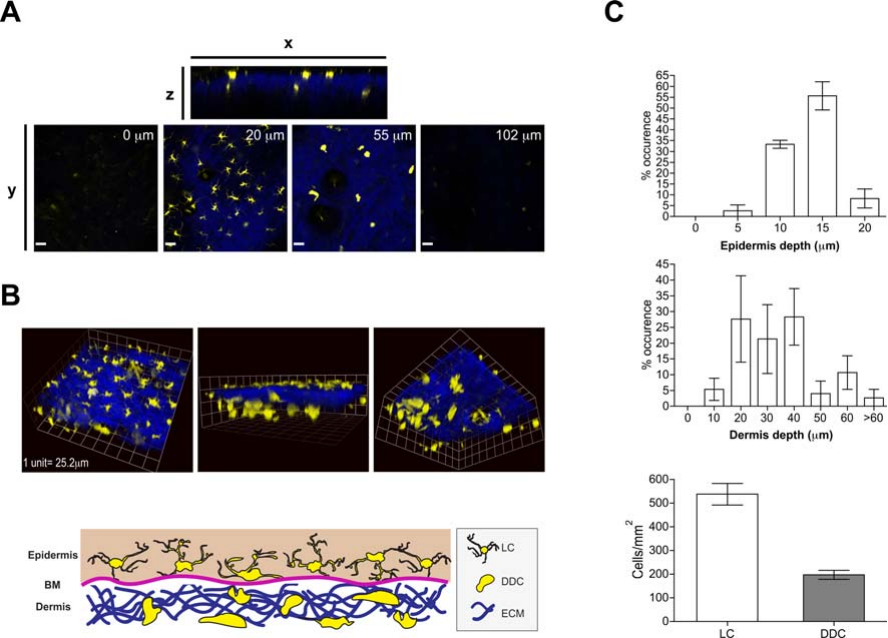
Three-dimensional distribution of dendritic cells within CD11c-YFP mice. (A) Single plane images from 2P-IVM showing YFP^+^ dendritic cells (yellow) in ear skin at various vertical depths along the z-projection. Extracellular matrix in the dermis was detected by the SHG signals (blue). Scale bar, 49 µm. (B) *Upper panels*, representative images from three-dimensional reconstructions of ear skin of a CD11c-YFP mouse showing the distribution of LC and DDC in relation to SHG. *Lower panel*, a schematic representation of DC localization in relation to different compartments in the skin. (C) Upper and middle histograms depict numbers of YFP^+^ LC and DDC along the vertical depth in the epidermis and dermis (underneath basement membrane). Lower histogram shows LC and DDC density in the epidermis and dermis (between 20–50 µm). Bars represent mean±SEM numbers obtained from at least three individual mice.

The distribution of YFP^+^ DC populations was determined by 2P-IVM in the ear skin of CD11c-YFP mice. Vertical scans revealed the presence of YFP^+^ cells between 5–20 µm below the outermost epidermal layer (Figure 2A–2C). These cells exhibited numerous, irregularly shaped dendrites, morphologically consistent with LC. The highest density of LC was found 15 µm underneath the stratum corneum (Figure 2C). Below the epidermis, second harmonic generation (SHG) signals highlighted extracellular matrix (ECM) components [21] forming a dense, mesh-like network (vertical depth of 20–60 µm from the outermost surface). Embedded in the lower part of this network, with the highest density between 20–40 µm below the basement membrane and reaching up to a depth of ∼100 µm, were scattered YFP^+^ cells, of markedly different morphology to LC, i.e. of rounder shape, with fewer, shorter dendrites (Figure 2A–2C). The overall density of LC was approximately 3 times higher than that of DDC (Figure 2C). Together, these results established that cutaneous skin DC populations could be imaged by means of 2P-IVM, and identifed two morphologically distinct cutaneous DC subsets in the different compartments of the skin.

### DDC Migrate through the Interstitial Space in a Gα_i_ Protein-Coupled Receptor–Dependent Manner

**Figure 3:**
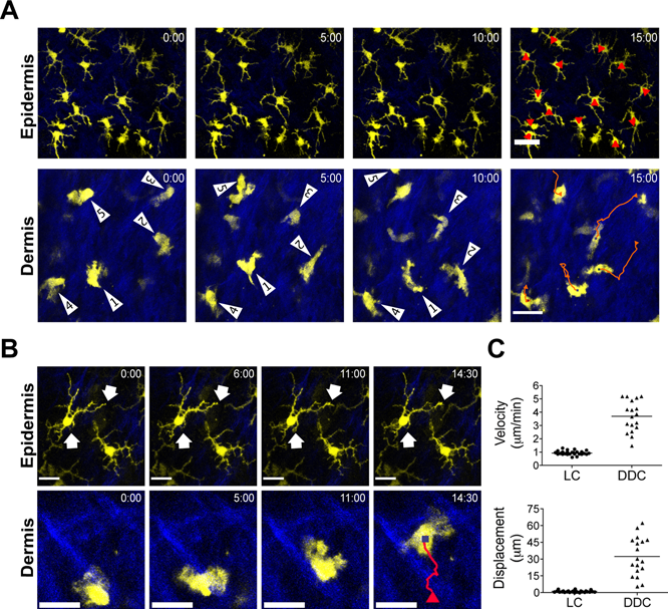
Migratory behavior of LC and DDC. (A) Representative time-lapse images from 2P-IVM showing the migratory behavior of LC and DDC. Red line, track of migration during the observation period. Scale bar, 25 µm. (B) Representative high magnification time-lapse images showing the cellular movement of LC and DDC. Scale bars, 16 µm (epidermis) and 25 µm (dermis). Arrows illustrate dendrite movements. Red line, track of migration during the observation period. (C) *Upper panel*, mean velocity of LC and DDC; *lower panel*, displacement of LC and DDC from 15 min tracks. Symbols represent individual cells.

While epithelial DC populations in the skin and gut have been found to be sessile [19],[22], no information is available on DC behavior in the interstitial space within peripheral organs. Nevertheless, DC in the dermis are suspected to be involved in antigen transport from the skin to draining LN thereby regulating the initial phases of host-pathogen responses. We therefore asked whether DDC scanned their microenvironment in a similar fashion to epidermal LC. To this end, we conducted time-lapse 2P-IVM in ear skin of CD11c-YFP mice. When focusing on the epidermis, we found that LC were sessile (mean velocity <2 µm/min), with their dendrites remaining almost completely immobile (Figure 3A–3C and Video S1). As described previously, we occasionally observed dSEARCH [4],[8] (Figure 3B and Video S2). However, in contrast to LC, we discovered that DDC were actively crawling through the interstitial space of the dermis at a mean velocity of 3.7±0.3 µm/min (mean±SEM) (Figure 3A–3C and Video S3). Migrating cells exhibited a polarized morphology, often displaying lamellipodia at the leading edge and a trailing uropod-like structure (Video S3). Since our experiments were performed in non-inflamed ear tissue, these results suggest that continuous migration is a steady-state feature of interstitial cutaneous DC. It may further indicate that the unexpectedly high motility of DDC serves to screen the dermal extracellular space for intruding microorganisms/environmental noxae.

**Figure 4:**
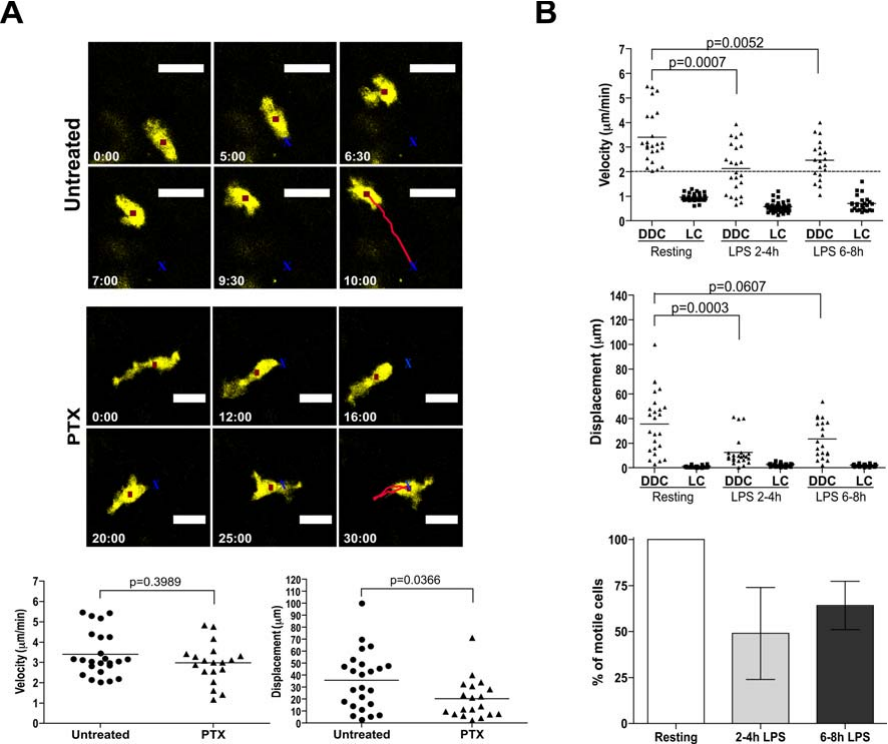
Migratory mechanisms of DDC and LC. (A) Response of DDC to systemic injection of PTX. *Top* and *middle panels* depict a representative cell under control and PTX treatment conditions, respectively. The red square indicates the cell centroid, and the red line shows movement of the centroid over the observation period (n = 3 experiments for PTX treatment). *Lower panel*, data points represent individual cells, lines indicate mean. (B) Upper and middle plots show mean velocity and displacement of LC and DDC in response to systemic LPS challenge over time (n = 3 experiments). Data points represent individual cells, lines indicate mean. Lower plot shows the frequency of motile DDC at resting state, 2 to 4 h and 6 to 8 h after LPS treatment from 30 min tracks (bars represent mean±SEM).

We next sought to define signals involved in spontaneous migration of DDC. When we treated animals with pertussis toxin (PTX), an inhibitor of Gα_i_ protein-coupled receptors, the capability of DDC to translocate within the dermis significantly decreased (reflected by a reduction of their displacement; Figure 4A and Videos S4 and S5). The migratory velocity of DDC did not change after PTX treatment, because cells moved back and forth in the same place (therefore, following cell-centroids resulted in measurable velocity; Figure 4A and Video S5). We concluded that, while PTX does not interfere with the migratory machinery of DDC *per se*, DDC utilize chemo-attractant signals, most likely chemokines, for their migration through the interstitial space.

### Encounter of Danger Signals Leads to Migratory Arrest of DDC

Having defined the cellular activities of skin DC in the steady-state, we determined their behavior in the presence of danger signals implicated in DC activation [23]. CD11c-YFP mice were injected intravenously with LPS (50 µg), which mimics systemic bacterial infection [24]. Two to eight hours after LPS treatment, LC remained sessile within the epidermis, without evidence of lateral or vertical movement (Figure 4B). In contrast, we observed dramatic changes of DDC behavior two to four hours after LPS administration. They exhibited significantly decreased migratory velocity (2.12±0.21 µm/min) and displacement, with more than 50% immobile cells (Figure 4B, Figure S2 and Video S6). Six to eight hours post LPS injection DDC partially regained their mobility (70% motile cells; Figure 4B, Figure S2, and Video S7). Thus, upon encounter of danger signals, DDC change their behavior, which may facilitate recognition/uptake of pathogens.

### DDC Rapidly Take Up *L. major* Promastigotes after Intradermal Deposition

To further test this hypothesis, we used the protozoan parasite *L. major* as a model pathogen. During natural infection, promastigote stage *Leishmania spp*. are directly deposited into the dermis by sand flies. Previous *in vitro* studies have demonstrated that DC can be infected by *Leishmania* parasites [13],[25]. We therefore speculated that DDC may recognize and interact with *L. major* upon introduction into the dermis.

**Figure 5:**
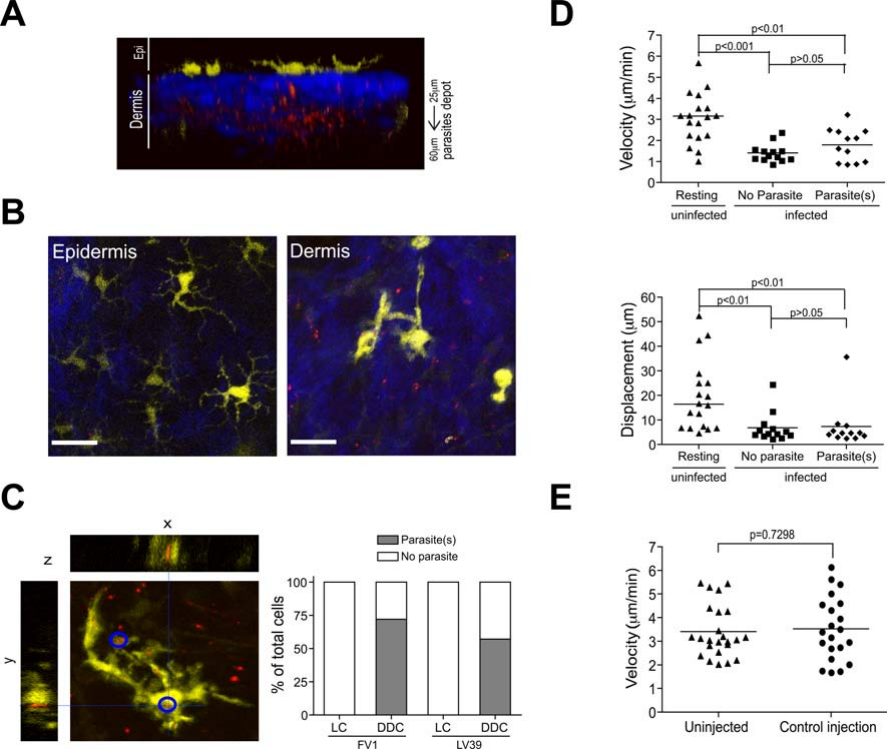
Internalization of *L. major* by DDC. (A) Three-dimensional reconstructions of ear skin inoculated with LmjF-DsRed2 promastigotes (red) showing the distribution of parasites (90 serial optical sections, 1 µm step size). (B) Representative images showing the morphology of LC (epidermis, yellow) and DDC (dermis, yellow) after LmjF-DsRed2 promastigote (red) inoculation. (C) A three-dimensional section of DDC (yellow) containing intracellular LmjF-DsRed2 promastigotes (red). Plot shows the frequency of LC and DDC containing LmjF or LV39 parasites (>50 cells obtained from randomly selected fields). (D) Comparison of the mean velocity and displacement of DDC in control skin, and DDC in infected skin with or without internalized parasites. Data points represent individual cells, lines indicate mean. Data were obtained from at least three independent experiments. (E) SNARF-1 was injected i.d. and DDC migration determined after 2 h (n = 3 experiments). Symbols represent individual cells. Control data are the same as in Figure 4A.

1–2×10^5^ DsRed2-tagged *Leishmania* (LmjF-DsRed2) promastigotes were injected in a small volume (1.5 µl) of saline solution into the superficial dermis. This allowed us to deposit parasites underneath the epidermis at a vertical depth of 25–60 µm while keeping mechanical tissue disruption as minimal as possible (Figure 5A). Within 20 min of injection, DDC in the vicinity of parasites decreased their migratory speed and changed their shape to a more dendritic cell-like morphology characterized by the emergence of multiple dendritic processes (Figure 5B and 5C). This was paralleled by the appearance of several intracellular vacuoles, each of them containing a single red parasite (Figure 5C), which is consistent with the formation of PVs [26],[27]. Interestingly, these vacuoles were mobile, i.e. appeared to move freely within the cytoplasm of the cells. Two to three hours after infection, the percentage of DDC harboring one or more parasite was ∼70% (Figure 5C). Of note, LC morphology and behavior was unchanged after infection with *L. major*. Further, LC were not found to take up parasites, at least during the first six hours of infection (data not shown). However, it should be pointed out that parasites were injected intradermally. Consequently, LC access to parasites may have been prevented by anatomical barriers, such as the epidermal basement membrane.

To determine whether parasite uptake by DDC was specific for the Friedlin strain (FV1) of *L. major*, or could be recapitulated with other *L. major* strains, we injected the LV39 strain under the same conditions as described above. As shown in Figure 5C, this led to ∼55% of DDC containing parasites. We therefore consider *L. major* uptake by DDC a general phenomenon.

For most of our experiments we made use of stationary phase *L. major* promastigotes. Since these cultures may contain stages of different infectivity or even a few dead parasites, confirmatory experiments (n = 3) using highly purified metacyclic [28] LmjF-DsRed2 parasites were conducted. These experiments confirmed uptake of parasites by YFP^+^ DDC into cytoplasmic vacuoles to the same extent as stationary phase parasites (Figure S3).

### Local *Leishmania* Infection Leads to Migratory Arrest of Both Infected and Uninfected DDC

Since our LPS experiments had shown that DDC markedly reduce their locomotion after exposure to a danger signal, we next assessed the migratory behavior of *L. major*-bearing DDC. As shown in Figure 5D, infected DDC significantly reduced their migratory velocities. To determine whether parasite uptake and migratory arrest were related phenomena, we also measured the migratory speed of non-infected DDC. We found that the latter revealed a similar reduction in their migratory velocities and displacement as compared to their infected counterparts. Collectively, these results show that DDC, by default, reduce their migration at sites of inflammation.

We also conducted sham infection experiments using a red fluorescent dye, SNARF-1, in order to exclude that the physical manipulation during intradermal injection by itself caused changes in DDC behavior. As shown in Figure 5E, there was no difference in DDC migration between SNARF-1 injected and uninjected skin attesting to the specificity of the infection experiments.

### Uptake of *L. major* by DDC Occurs through Motile Pseudopods

**Figure 6:**
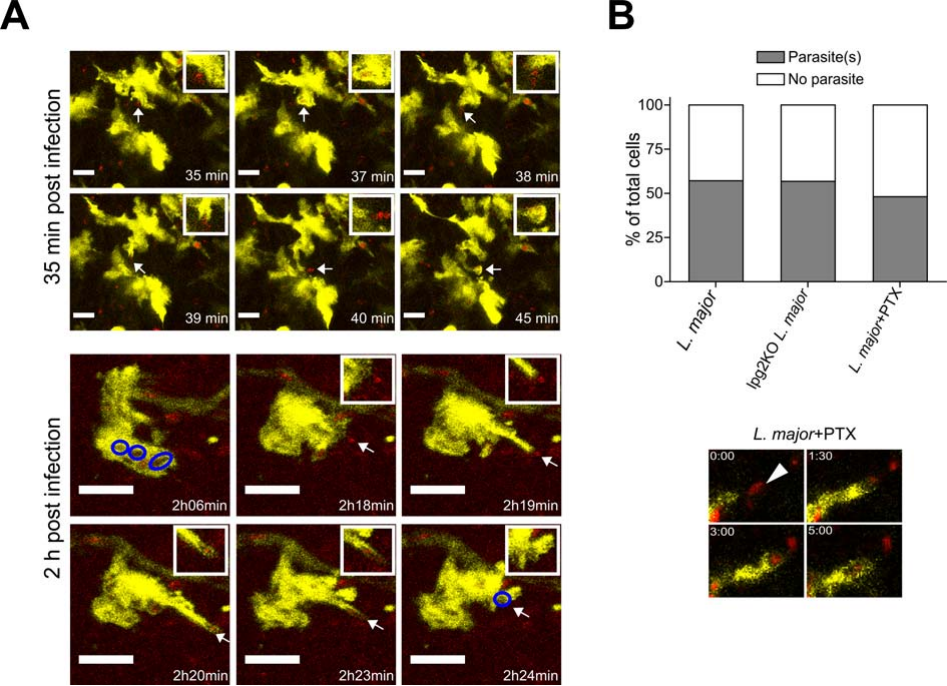
DDC extend pseudopods to engulf *L. major* parasites. (A) Representative time-lapse images showing uptake of parasites (red) by rapid extension/retraction of pseudopods from DDC. Scale bars, 12 µm (upper panels) and 6 µm (lower panels). Small inlet shows tip of pseudopod at high magnification. Blue circles illustrate parasite-containing vacuoles. (B) Graph shows the effects of systemic PTX treatment on LmjLV39-DsRed2 parasite uptake by DDC in close vicinity to a parasite depot (n = 3 experiments). Images depict high magnification of parasite uptake by a dendrite after PTX treatment. Also shown is the uptake of lpg2KO-DsRed parasites by DDC (n = 3 experiments).

The exact mode by which *Leishmania* infects cells *in vivo* is not known. It is thought that parasite uptake by phagocytes involves non-random promastigote attachment to the cell followed by engulfment [29]. However, only *in vitro* data on this process are currently available, and the cellular and molecular mechanisms remain poorly understood. Our intravital imaging experiments demonstrated that cytoplasmic DDC processes actively extended towards parasites (Figure 6A and Videos S8 and S9) at an average speed of ∼2.5 µm/min and reaching up to 50 µm in length. We occasionally observed that dendrite extension was preceded by parasite contact with the cell membrane followed by engulfment along the long axis of the parasite (Figure 6A and Videos S8 and S9). After capturing parasites, dendrites often rapidly retracted towards the cell body, paralleled by the formation of an intracellular vacuole. These results establish that *L. major* parasites in the interstitial space were internalized in a free form by DDC during the early phase of infection.

We next asked whether inhibition of Gα_i_ protein-coupled receptor signaling interfered with parasite uptake by inoculating mice with LmjLV39-DsRed2 parasites two to three hours after systemic PTX treatment. Since after PTX application DDC did not translocate through the dermis, we imaged cells that co-localized with the parasite depots. We observed that the formation of pseudopods and parasite uptake was preserved in these non-displacing DDC (Figure 6B). This indicates that parasite sensing was independent of Gα_i_ protein-coupled receptors. Furthermore, these results show that migration and dendrite formation can be uncoupled at the molecular level.

### Phosphoglycans Are Not Involved in Parasite Interactions with DDC

Phosphoglycans (PG), in particular lipophosphoglycans (LPG), are essential during the infectious cycle of *Leishmania*. For instance, PGs have been implicated in the adherence of parasites to the gut epithelium in the sand fly, the resistance to complement in the blood stream, and have been considered candidate molecules for the uptake by host cells [13],[30]. PG-deficient parasites persist *in vivo* for months without causing disease, and are therefore considered potential attenuated anti-*Leishmania* vaccine candidates [31]. While *in vitro* studies have shown that macrophages can take up PG-deficient parasites, it is not known whether the target cell range is the same for PG-deficient and wildtype parasites *in vivo*. To gain further insight into the role of LPG in parasite interactions with DC *in vivo*, we made use of DsRed2-tagged *L. major* deficient in the LPG2-encoded Golgi GDP-mannose transporter. These parasites fail to synthesize surface and other secreted PG [32]. As shown in Figure 6B, lpg2KO-DsRed2 parasite uptake was similar to that of LmjLV39-DsRed2 control parasites. Therefore, PGs appear to be dispensable in the initial sensing of parasites by dendrites as well as in the binding of parasites to the cell membrane and subsequent internalization.

### DDC Discriminate between *L. major* Parasites and Inert Material

**Figure 7:**
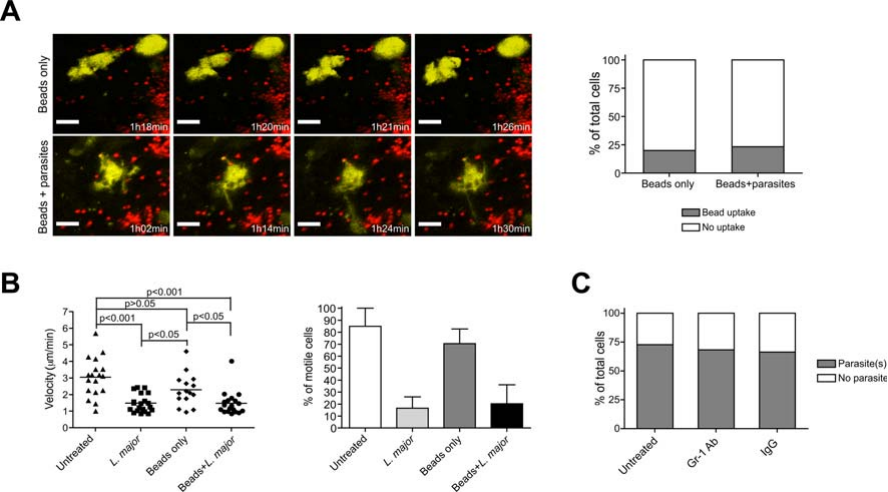
Discrimination of inert beads and *L. major* uptake by DDC and role of neutrophils in parasite uptake. (A) *Left panels*, time-lapse images from 2P-IVM showing the cellular behaviors of DDC (yellow) after fluorescent bead inoculation alone (red) or beads (red) together with *L. major* promastigotes (unlabelled). *Right panel*, frequency of DDC with intracellular beads in the presence or absence of *L. major* parasites (>50 cells obtained from randomly selected fields). Scale bar, 12 µm. n = 3 experiments. (B) Mean velocity and frequency of motile DDC (mean±SEM ) at resting state, after LmjF-DsRed2 promastigote, beads only, and beads plus *L. major* promastigote inoculation. Data points represent individual cells. (C) Frequency of DDC with internalized LmjF-DsRed2 parasites in the ear skin of control (IgG), or neutrophil depleted (Gr-1 Ab) CD11c-YFP mice. n = 3 experiments.

DC can, in principle, internalize a large variety of particulate material [33]. Thus, we next determined whether parasite uptake was a specific phenomenon, or whether DDC indiscriminately incorporate particles introduced into the dermis. When inert fluorescent beads were injected intradermally, a minority (∼20%) of DDC revealed intracellular beads at a low number (usually 1 bead/cell) two to four hours after injection (Figure 7A, Table 1, and Videos S10 and S11). When counting the ratio between particles present in the immediate vicinity of DDC (i.e. within half a cell diameter) and intracellular particles, it was obvious that there was a clear preference of *L. major* uptake (ratio 2.7) as compared to bead uptake (ratio 39.5; Table 1). In addition, we never observed dendrite formation after bead injection.

**Table 1:** *L. major* and bead uptake by DDC.

Cell	*L. major*		Beads	
	Extracellular	Intracellular	Extracellular	Intracellular
1	3	1	6	0
2	2	1	3	0
3	1	0	16	1
4	5	8	8	1
5	10	2	10	0
6	5	0	7	0
7	4	1	11	0
8	9	2	10	0
9	6	3	4	0
10	3	0	4	0
**Total**	48	18	79	2
**Mean**	**4.8**	**1.8**	**7.9**	**0.2**
	Ratio = 2.67		Ratio = 39.5	

Two to four hours after *L. major* or bead injection, individual DDC (number 1 to 10) were randomly selected, and the number of the respective particles present intracellularly or within half a cell diameter around individual DDC determined (n = 10 DDC) from 3 individual experiments. Ratio equals mean extracellular:mean intracellular particles.

The bead injection procedure did not result in significant changes in migratory velocity or shape change of the cells (Figure 7B), indicating that the mechanical trauma induced by the inoculation was not sufficient to alter DDC behavior. However, it was conceivable that an inflammatory stimulus may have increased phagocytic activity of DDC. To test this further, we co-injected beads and parasites. Interestingly, there was no increase in bead incorporation, demonstrating that DDC are capable of selectively discriminating between *L. major* parasites and inert material.

### Parasite Uptake by DDC Is Independent of Neutrophils

Finally, we determined whether *L. major* uptake was a primary feature of DDC or was facilitated by other innate immune cells present in early infection. In particular neutrophils have been shown to serve as vectors for *Leishmania* uptake by macrophages [34]. Depletion of these cells prior to intradermal injection of LmjF-DsRed2 did not change the number of DDC containing parasites as compared to controls (Figure 7C). While these results do not exclude a role of neutrophils in the defense against this pathogen, they suggest that parasite phagocytosis by DDC is independent of these cells.

## Discussion

DC are considered gatekeepers in the defense against intruding pathogens. While DC responses to microbes have been studied in great detail *in vitro* and in cells isolated *ex vivo*, very little is known about their interactions in the context of intact tissues in real time. The present study aimed to visualize, in a dynamic manner, the behavior of DDC in normal skin and in response to a defined pathogen. Using 2P-IVM, we found that, under homeostatic conditions, DDC were actively crawling through the dermal interstitial space. Remarkably, upon introduction of the protozoan parasite *L. major*, DDC transformed into stationary, dendritic-shaped cells that were capable of rapid parasite uptake through flexible dendritic processes. Together, our findings define the microenvironmental context of DDC-pathogen encounter in the earliest phase of cutaneous immune responses.

That DDC migrate in the steady-state was unexpected, as other DC populations, such as DC in the gut epithelium and the epidermis have been found to be immobile, or in the case of the T cell area, very slow moving [4],[8],[19],[22]. It is likely that the specific cellular motility patterns adopted by these individual DC populations serve to optimize their functions in their respective microenvironments. For instance, epidermal LC are in continuous close contacts with surrounding keratinocytes. The paucity of extracellular space may not require, or may not allow, movement of the cells for their immunosurveillance function. Rather, soluble antigens percolating through the extracellular epidermal space or signals from neighboring keratinocytes and/or adjacent LC may be sensed by the communicating dendrite network in this environment. DC in the LN T cell zones are characterized by extensive motions of their dendrites, which may be important for sensing of antigens filtering through the conduit system of this organ, and for establishing contacts with naïve T cells [19]. As compared to the epidermis, DDC are localized within the much more extensive dermal space, which, at the same time, contains considerably lower densities of resident cells, primarily fibroblasts. Thus, while keeping in mind that other tissue resident cells were not visualized in our study, DDC appeared as isolated cells embedded within the network of dermal ECM fibers. They were also found to be morphologically distinct from LC, i.e. they did not exhibit dendrites under non-inflammatory conditions. Therefore, the observation of their continuous crawling indicates a fundamental difference in tissue screening as compared to LC as well as DC in the T cell areas of LN. Since signals from intercellular communication by DDC with other dermal cells may be less abundant than for LC in the epidermis or DC in LN T cell areas, spontaneous DDC migration guarantees access to every corner of this specific microenvironment regardless of the activation state or potential damage of other resident cells during infection. Consequently, this ensures the rapid detection of intruding microbes and the subsequent immediate response to danger signals.

Morphologically, DDC appeared to migrate in an amoeboid fashion, similarly to what has been described for T cells in the extravascular space [35],[36]. Thus, crawling DDC exhibited an anterior-posterior asymmetry reflected by the formation of lamellipodia and uropods. This may suggest that similar molecular cues responsible for interstitial T cell migration, for example surface receptors involved in communication with the environment as well as intracellular molecules, mediate DDC locomotion. We found that blocking of Gα_i_ protein-coupled receptors with PTX significantly reduced the displacement of DDC, implying chemoattractant receptors, such as chemokines or lipid mediators, in this process. This is consistent with recent 2P-IVM studies demonstrating that PTX inhibited the migration of naïve T cells within the LN paracortex, and that CCR7 is, at least partially, involved in this process [37]–[39]. However, the T cell zone of LN contains the fibroblastic reticular cell (FRC) network, which provides the structural backbone of this particular microenvironment. Elegant imaging experiments by Germain's group have shown that the FRC network acts as a scaffold for migrating naïve T cells [40]. A similar cellular structure does not exist in the dermis, raising the question as to how migrating DDC orient themselves within the dermis. It is conceivable that interactions with the extracellular matrix, primarily collagen fibers, are responsible for this process. Indeed, high resolution imaging has shown the intimate contact between DDC and the ECM (Figure 3), and it is likely that chemoattractants are deposited along these structures. Future studies will address the role of specific adhesion receptors, such as integrins or the hyaluronan receptor CD44, as well as specific chemoattractant receptors in these interactions.

The fact that DDC seemed to survey the dermis made us wonder whether they were indeed capable of detecting microorganisms introduced into the dermis. We chose the protozoan parasite *L. major* as a model pathogen, which is ideal in this context because, firstly, the parasite is directly deposited in the dermis during natural infection by sand flies, and secondly, the parasite is of sufficient size to be detected by 2P microscopy both extra- and intracellularly. Furthermore, the innate and adaptive immune responses against *Leishmania spp*. have been characterized in great detail in the past, even though controversy exists as to whether DC themselves are infected by the parasite during early infection (reviewed in [11],[13],[25]). While the exact number of parasites transferred during sand fly bites is not known, inoculation of as few as 100 metacyclic parasites is sufficient for establishing an infection [41]. Although we could observe parasite uptake by YFP^+^ DDC by injecting as few as 2×10^4^ parasites (data not shown), this was technically challenging as only very few parasites and DC could be visualized *in situ* when using such low numbers. Thus, for the experiments in the present paper, 1–2×10^5^ parasites were used in order to obtain data for proper statistical analysis. It should further be pointed out that the use of small volumes (in the range of 1–2 µl) for intradermal injection was critical, as larger volumes (particularly >5 µl) resulted in the disruption of the local microenvironment. This was evidenced by a disturbance of ECM fibers due to excess fluid (edema) and a migratory decrease/arrest of DDC within the injected ear, even after injection of saline solution without an inflammatory stimulus (data not shown). In contrast, using our injection protocol, we did not observe a migratory or morphological change of DDC imaged ∼50–200 µm away from the injection site under control conditions (Figures 5E and 7B). This result bears consideration not only for imaging studies, but for any situation in which the function of DDC is studied.

In our intradermal infection model, we found that the majority of DDC picked up one or more *L. major* parasites shortly after inoculation. This was consistent when using two independent *L. major* strains, supporting the hypothesis that DDC are indeed capable of detecting this protozoan parasite *in vivo*. Interestingly, after the introduction of parasites, DDC underwent a morphological transition into *bona fide* DC-shaped cells. Strikingly, parasites appeared to be taken up by long, motile pseudopods (Videos S8 and S9). *In vitro* infection models of macrophages demonstrated that parasites initially adhered to the cell membrane in a non-random orientation, i.e. preferentially with either the tip or the base of their flagellum [29]. Subsequently, the parasites were engulfed by pseudopods wrapping around the parasites (“coiled phagocytosis” [42]). We found that dendrite extension was sometimes preceded by parasite contact with the cell membrane, while at other times no visible contact was obvious. However, the level of 2P-IVM resolution did not always allow for unequivocal visualization of the parasite flagellum. Therefore, it is conceivable that physical contact is the main trigger of DDC dendrite extension observed in the context of *Leishmania* infection.

Recently it has been suggested that DDC are composed of two separate subpopulations, i.e. the major langerin^−^ subset and a small langerin^+^ subset [43]–[45]. Langerin^+^ DDC are distinct from in-transit LC, and have been shown to be capable of inducing cutaneous hypersensitivity reactions independently from langerin^−^ cells. However, these cells are very rare (2–10% of DDC), and it is unclear whether their functions are different to langerin^−^ cells. Since in our experiments 55–70% of all DDC are infected by *L. major*, the vast majority will represent langerin^−^ cells. Together with previous studies showing that langerin^−^ DC in draining LN present *Leishmania* antigens to T cells, we therefore speculate that langerin^−^ DDC are the major players in this scenario. Ablation of langerin^+^ DDC using genetic approaches will enable definitive answers to this question.

What are the mechanisms of parasite recognition by dendrites? In the intestine, subepithelial DC have been found to extend processes between epithelial cells into the gut lumen, often revealing a spherical shape (“balloon bodies”) [22],[46]. While these processes were capable of capturing bacteria in the gut lumen in a passive manner, this appeared to be a rare event. Importantly, sampling of gut material was non-discriminatory, i.e. DC did not distinguish between inert beads and bacteria [22]. In our study, we found that inert material (beads) alone or co-injected with parasites was largely ignored by DDC. In addition, when we injected fluorescently-tagged Bacillus Calmette-Guérin (BCG), we found that two to four hours after inoculation only ∼30% of DDC contained single internalized BCG, comparable to the results using beads (Figure S4 and data not shown). Furthermore, under these conditions we did not observe the transition of DDC into highly dendritic cells, even when they contained bacteria (Figure S4 and data not shown). Together, these results suggest that *L. major* induces a specific change in DDC *in vivo* (i.e. pseudopod formation), and may indicate the involvement of specific surface receptor(s) in this process. Previous studies have shown that Fc receptors and complement receptors are involved in *Leishmania* uptake by phagocytic cells. However, the molecular cues recognized on parasites are not well understood. Our studies have shown that PGs are not involved in parasite uptake by DDC. The use of parasite strains deficient in a variety of other structural and metabolic genes may, in the future, identify the molecular requirements of parasites to be sensed by dendritic processes.

Another key observation from this study was the rapid transformation of migratory DDC into sessile DDC after exposure to microbial products, such as LPS. In addition, both infected and uninfected DDC became non-migratory at sites of *L. major* injection, suggesting that the inflammatory environment induces the change in migratory behavior, rather than parasite uptake *per se*. This conceivably also reflects a switch in functionality of DDC, i.e. from surveillance to sampling/antigen uptake. Thus, by arresting DDC in close proximity to the site of infection, they form a network of sessile cells “primed” for uptake of microbes present at the site. That these states are indeed distinct from each other is further reflected by the fact that PTX treatment interfered with DDC translocation, but not with parasite uptake. These findings raise the question as to the fate of DDC infected early during parasite infection. We have noted that DDC loaded with *Leishmania* remained relatively sessile over a period of up to ∼6 hours post-inoculation (unpublished observation). When imaging at later time points (∼20 hours post infection), there were numerous YFP^+^ cells present within the dermis. While these cells showed a similar non-migratory phenotype as cells at earlier timepoints, the number of parasite-containing DDC decreased (unpublished observation). Nevertheless, parasites were still present in the dermis, presumably within other cells (unpublished observation). This may suggest that infected DDC leave the dermis at this stage in order to migrate to draining LN, and that these cells may be replaced by newly immigrating DC or their precursors from the bloodstream. Indeed, previous studies have shown that infected DC arrive in draining LN around 24 hours after infection [5]. Alternatively, parasites within DDC *in vivo* may simply lose fluorescence over time possibly due to an inability to survive for prolonged periods of time within these cells. Future studies will address potential interactions of infected DDC with the lymphatic vasculature in the dermis, and how these interactions are regulated at the molecular level.

## Materials and Methods

### Reagents

Anti-mouse CD11b, CD11c, CD45.2, F4/80, I-A^b^ (all from BD Biosciences), Langerin (Dendritics, Lyon, France) and Moma-2 (Abcam, Cambridge, UK) antibodies were used for flow cytometry analysis of epidermal and dermal cell suspensions.

### Animals, *L. major* Parasites, and BCG Strain

CD11c-YFP mice [19] (kind gift of Dr. Michel Nussenzweig) on a C57BL/6 background (10 generations) were bred in the animal facility of the Wistar Institute and the Centenary Institute. Animal experiments were performed with approval of the Institutional Animal Care and Use Committees at both institutions. To generate fluorescent protein expressing *L. major* parasites, the gene encoding the red fluorescent protein DsRed2 was PCR amplified from pDsRed2 (Clontech) with primers that added BamHI sites to both ends. The PCR product was cut with BamHI and ligated into BglII site of pIR1SAT yielding pIR1SAT-DsRed2 (strain B4787). After SwaI digestion, it was introduced into *L. major* strain Friedlin V1 (MHOM/IL/80/Friedlin) by electroporation [47]. DsRed2-expressing LV39 clone 5 (Rho/SU/59/P) and its *LPG2*-deficient derivative were generated by stable transfection of Swa I-cut pIR1-SAT-LUC-DsRed2 (B5947). This plasmid was obtained by ligating the NruI-SalI DsRed2 fragment from pIR1SAT-DsRed2 (B4787) into SalI+NruI digested pIR1SAT-LUC (B5037). Clonal transfectants were obtained and screened for bright red fluorescence and virulence in mouse infections (data not shown). One clone of each strain was selected for work here (*L. major* FV1 SSU:IR1SAT-DsRED2(b), LmjF-DsRed2; LV39 SSU:LUC:DSRED2, LmjLV39-DsRed2; LPG2KO SSU:LUC:DSRED2, lpg2KO-DsRed2). Promastigotes were grown in complete M199 as described previously [47]. Red fluorescent protein expressing BCG was generated by transforming BCG Pasteur with plasmid pSMT3:mCherry (a kind gift of Dr Wilbert Bitter, VU University Medical Center, Amsterdam, the Netherlands) as previously described [48]. Hygromycin-resistant colonies were selected on Middlebrook 7H11 medium (Difco Laboratories, Detroit, MI, USA) and expanded in liquid Middlebrook 7H9 medium. Fluorescent colonies were selected by flow cytometry.

### Preparation of Epidermal and Dermal Cell Suspensions

Epidermal and dermal cell suspensions were prepared as described previously [49] with some modifications. In brief, ear tissues were incubated in trypsin (0.5%) in HBSS buffer (Invitrogen) for 1 h at 37°C. For CD11c staining, we made use of dispase (5 U/ml) instead of trypsin. After enzyme incubation, epidermis was separated from dermis. To obtain single cell suspensions, epidermal sheets were passed through a wire mesh, and dermal sheets were further digested with collagenase D for 1 hour.

### Treatment Protocols

For Gα_i_ protein-coupled receptor inhibition experiments, CD11c-YFP mice were injected intravenously with PTX (30 ng/g bodyweight) in saline solution. For LPS experiments, CD11c-YFP mice were injected intravenously with 50 µg of LPS. 2P-IVM was performed at various time points after the injections. For neutrophil depletion, CD11c-YFP mice were injected i.p. with 250 µg of anti-Gr-1 antibody or rat IgG as control 24 hours before the inoculation of *L. major* promastigotes. Splenocytes from these mice were examined by flow cytometry to ensure the depletion of neutrophils at the end of imaging (data not shown). In order to reduce autofluorescence from hairs, hair was removed from the ears for all imaging experiments [50]. Control experiments without the use of hair remover revealed identical migratory behavior of DDC (Figure S5).

### Intradermal *Leishmania* Parasite, Bead, and BCG Inoculation

Mice were anesthetized by intraperitoneal injection of Ketamine/Xylazine (80/10 mg/kg). 1–2×10^5^ stationary phase promastigotes in 1.5 µl of saline solution were injected intradermally using a 33 gauge Hamilton syringe. This procedure was performed under a stereoscopic microscope. For the bead experiments either 2.5×10^5^ FluoSphere microspheres (2 µm, Invitrogen) or 2.5×10^5^ microspheres plus 2.5×10^5^ FVI LmjF parasites were injected. For the BCG experiments, ∼2×10^5^ BCG were injected intradermally. As an additional control, the fluorescent dye SNARF-1 (10 µg/ml) was injected intradermally. Images were typically acquired 50–200 µm from the injection site.

### Two-Photon Intravital Microscopy and Image Analysis

Anesthetized mice were placed onto a custom-built stage to position the ear on a small metal platform for 2P imaging. The ear was immersed in saline/glycerol (70∶30, vol∶vol) and covered with a coverslip. The temperature of the platform was maintained at 36°C, while the body temperature was regulated at 37°C through a heating pad placed underneath the mouse. Two-photon imaging was performed on a Prairie Technology Ultima System or a LaVision Biotec TrimScope equipped with a 40× (NA 0.8) water immersion objective [35]. Both setups included four external non-descanned dual-channel reflection/fluorescence detectors, and a diode pumped, wideband mode-locked Ti:Sapphire femtosecond laser (Coherent Chameleon or Spectra-Physics Mai Tai HP). The ear skin was exposed to polarized laser light at a wavelength of 950–960 nm. Three-dimensional (x,y,z) images of the ear skin were acquired (2–5 µm spacing in z-axis over a total distance of 10–25 µm) every 30 s for a total observation period of 1–2 hours. Images acquired were then transformed into time sequence movies using Volocity software (Improvision). Mean migration velocities, cellular displacement, and confinement ratios (total length of track divided by distance between starting and end point) were manually tracked and calculated for 15′ or 30′30″ as described previously [35]. Measurements were typically performed on 31 or 62 consecutive frames of the video. Cells were defined as immobile if the mean velocity was less than 2 µm/min [19]. To quantify the number of DC with internalized parasites, beads or BCG, images from 3D reconstructions of inoculated skin were examined for the colocalization of red signals (*L. major*, beads or BCG) and yellow signals (DC).

### Statistical Analysis

For comparisons, the Student's *t* test (normally distributed) or the Mann-Whitney test (not normally distributed) or one-way ANOVA were used. A difference was considered significant if P<0.05.

## Supporting Information

Figure S1 Flow cytometric analyses of YFP expression in the dermal cell population of CD11c-YFP and wildtype mice. (A) Dot plots show YFP signals in dermal cells in relation to I-A^b^ expression. Mean Fluorescence Intensity (MFI) of YFP is indicated in the plots. A YFP^+^I-A^b-low^ population was present in both mouse strains and was thus considered autofluorescent. Phenotypic analysis revealed that these cells represent macrophages (see Figure 1). (B) Maximum intensity images from 2P-IVM showing CD11c-YFP and WT mouse ear skin (dermis). While YFP bright cells are clearly detectable in CD11c-YFP mice, no YFP signal was detected in wildtype animals. Blue signals indicate second harmonic generation.
(0.09 MB PDF)

Figure S2 Effects of LPS on DDC migration. Representative tracks of DDC after LPS treatment (out of 3 experiments). Scale bars 49 µm.
(0.21 MB PDF)

Figure S3 Internalization of highly purified metacyclic *Leishmania* parasites by DDC. A three-dimensional section of DDC (yellow) containing several LmjF-DsRed2 promastigotes (red). The blue cross/circles point out intracellular parasites.
(0.04 MB PDF)

Figure S4 mCherry-BCG uptake by DDC. 2 h after intradermal mCherry:BCG (red) inoculation (2×10^5^ bacilli), a three-dimensional section of the ear was taken by 2P microscopy. A DDC (yellow) containing one intracellular BCG is visible (white box highlights BCG). Note that the DDC does not transform into a dendritic shaped cell.
(0.38 MB PDF)

Figure S5 Hair removal does not influence the behavior of DDC in the skin. Experiments were performed with or without hair removal (n = 3 mice).
(0.10 MB PDF)

Video S1 Behavior of epidermal LC. A time-lapse sequence of maximum projection (21 µm stack) showing the *in vivo* dynamics of LC movement in CD11c-YFP ear skin. Note the occasional extensions and retractions of dendrities (dSEARCH). Time is shown as hh:mm:ss.
(1.46 MB MOV)

Video S2 Dendrite movement of epidermal LC. A time-lapse sequence of maximum projection (21 µm stack) showing dSEARCH at higher magnification. Time is shown as hh:mm:ss.
(3.14 MB MOV)

Video S3 Dermal DC migration. A time-lapse sequence of maximum projection (21 µm stack) shows the migratory patterns of DDC in CD11c-YFP mouse ear skin. Time is shown as hh:mm:ss.
(5.16 MB MOV)

Video S4 Dermal DC migration. A time-lapse sequence of maximum projection (21 µm stack) shows the migratory patterns of a representative DDC in CD11c-YFP mouse ear skin. Red square indicates cell centroid, red line tracks the centroid over the observation period.
(0.20 MB MOV)

Video S5 Effects of PTX on dermal DC migration. A time-lapse sequence of maximum projection (21 µm stack) shows the migratory pattern of a representative DDC in CD11c-YFP mouse ear skin after systemic PTX treatment. Red square indicates cell centroid, red line tracks the centroid over the observation period.
(0.37 MB MOV)

Video S6 The migratory pattern of dermal DC after LPS injection (2 h). A time-lapse sequence of maximum projection (21 µm stack) showing DDC movement dynamics in CD11c-YFP ear skin 2 h after intravenous injection of 50 µg of LPS. Time is shown as hh:mm:ss.
(3.19 MB MOV)

Video S7 The migratory pattern of dermal DC after LPS injection (6 h). A time-lapse sequence of maximum projection (21 µm stack) showing DDC movement dynamics in CD11c-YFP ear skin 6 h after intravenous injection of 50 µg of LPS. Time is shown as hh:mm:ss.
(4.17 MB MOV)

Video S8 Dermal DC interact with *L. major* parasites. A time-lapse sequence of maximum projection (24 µm stack) shows a DDC extending a pseudopod to pursue and capture a moving LmjF-DsRed2 promastigote in the ear skin of a CD11c-YFP mouse. Time is shown as hh:mm:ss.
(1.56 MB MOV)

Video S9 A DDC containing multiple vacuoles capturing an *L. major* parasite. A time-lapse sequence of maximum projection (24 µm stack) shows a DDC extending a pseudopod to pursue and capture a moving LmjF-DsRed2 promastigotes in the ear skin of a CD11c-YFP mouse. Time is shown as hh:mm:ss.
(0.60 MB MOV)

Video S10 Behavior of dermal DC in *L. major* and beads (red) injected ear. A time-lapse of maximum projection (24 µm stack) shows DDC movement dynamics in the presence of *L. major* parasites (unlabelled) and beads (2 µm, red). Time is shown as hh:mm:ss.
(1.24 MB MOV)

Video S11 Behavior of a dermal DC in ear inoculated with beads only. A time-lapse sequence of maximum projection (24 µm stack) shows DDC movement dynamics in the presence of beads (2 µm, red) only. Time is shown as hh:mm:ss.
(0.25 MB MOV)
