# Supplementary figures and images for: Migratory Dermal Dendritic Cells Act as Rapid Sensors of Protozoan Parasites
Source: PLoS Pathog. 2008 Nov 28;4(11):e1000222. doi: 10.1371/journal.ppat.1000222 (PMC2583051; doi:10.1371/journal.ppat.1000222)

**A**

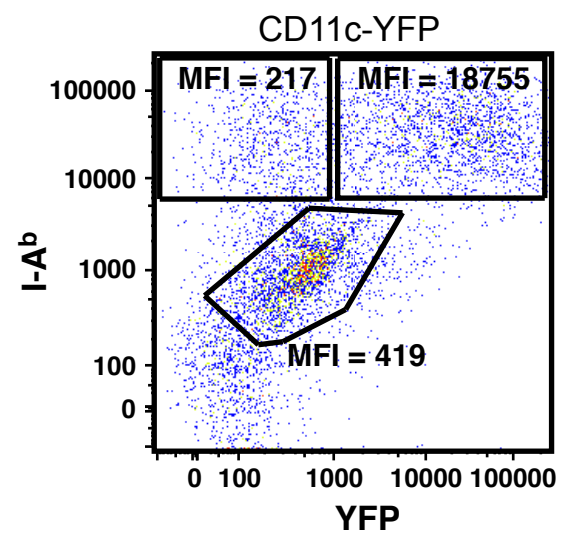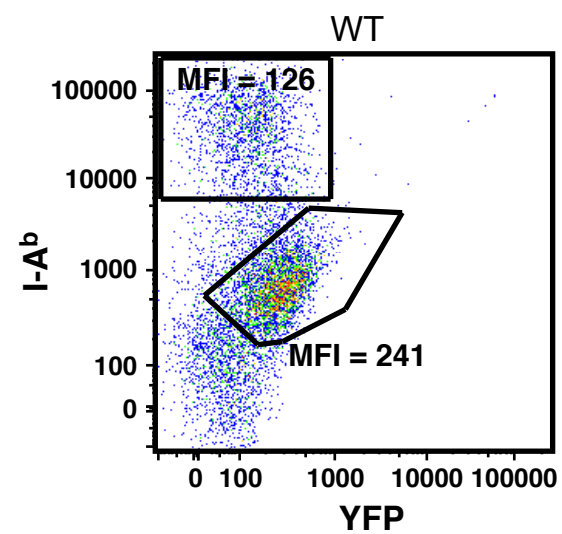

**B**

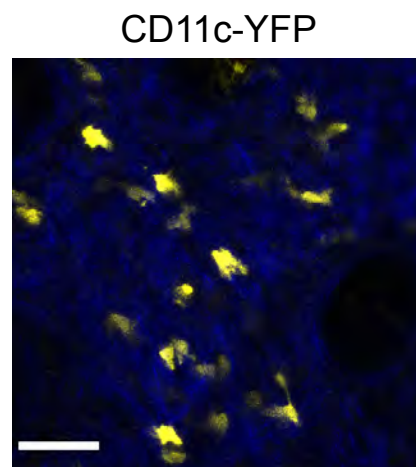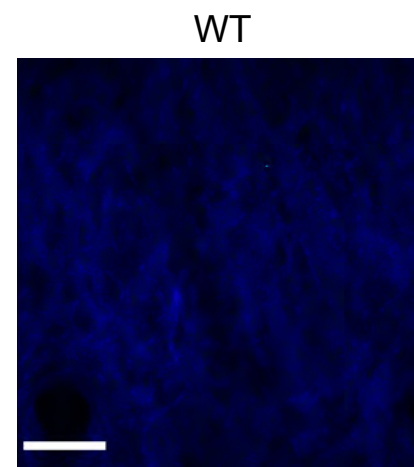

Supplement: Figure S1 — Flow cytometric analyses of YFP expression in the dermal cell population of CD11c-YFP and wildtype mice. (A) Dot plots show YFP signals in dermal cells in relation to I-Ab expression. Mean Fluorescence Intensity (MFI) of YFP is indicated in the plots. A YFP+I-Ab-low population was present in both mouse strains and was thus considered autofluorescent. Phenotypic analysis revealed that these cells represent macrophages (see Figure 1). (B) Maximum intensity images from 2P-IVM showing CD11c-YFP and WT mouse ear skin (dermis). While YFP bright cells are clearly detectable in CD11c-YFP mice, no YFP signal was detected in wildtype animals. Blue signals indicate second harmonic generation. (0.09 MB PDF) [file ppat.1000222.s001.pdf]

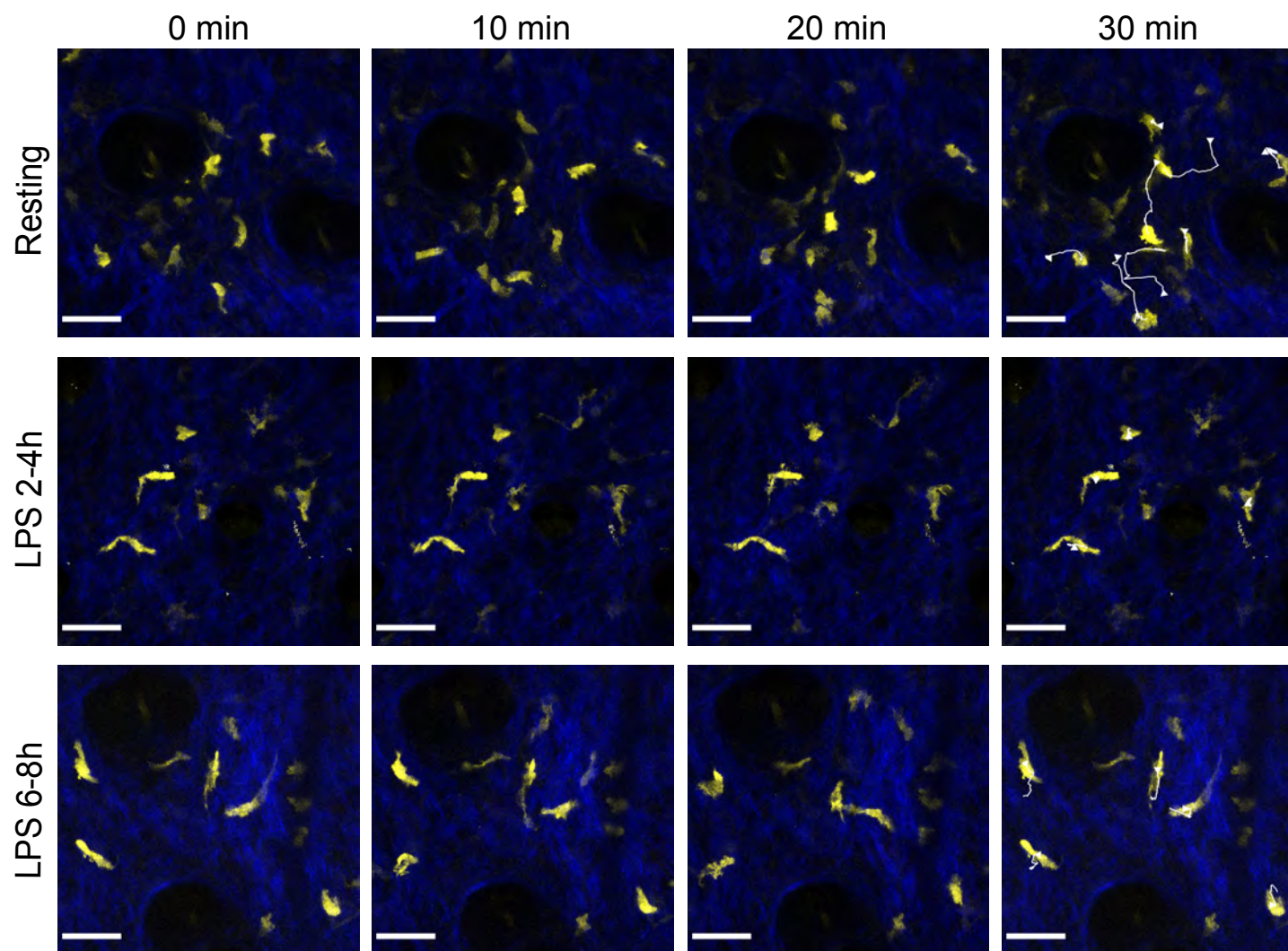

Supplement: Figure S2 — Effects of LPS on DDC migration. Representative tracks of DDC after LPS treatment (out of 3 experiments). Scale bars 49 µm. (0.21 MB PDF) [file ppat.1000222.s002.pdf]

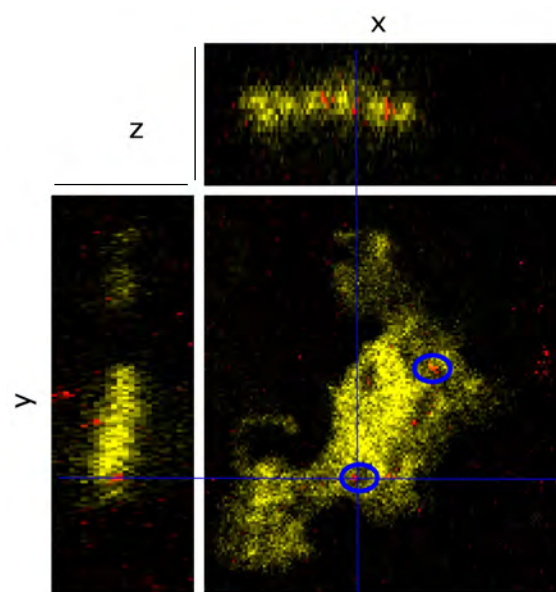

Supplement: Figure S3 — Internalization of highly purified metacyclic Leishmania parasites by DDC. A three-dimensional section of DDC (yellow) containing several LmjF-DsRed2 promastigotes (red). The blue cross/circles point out intracellular parasites. (0.04 MB PDF) [file ppat.1000222.s003.pdf]

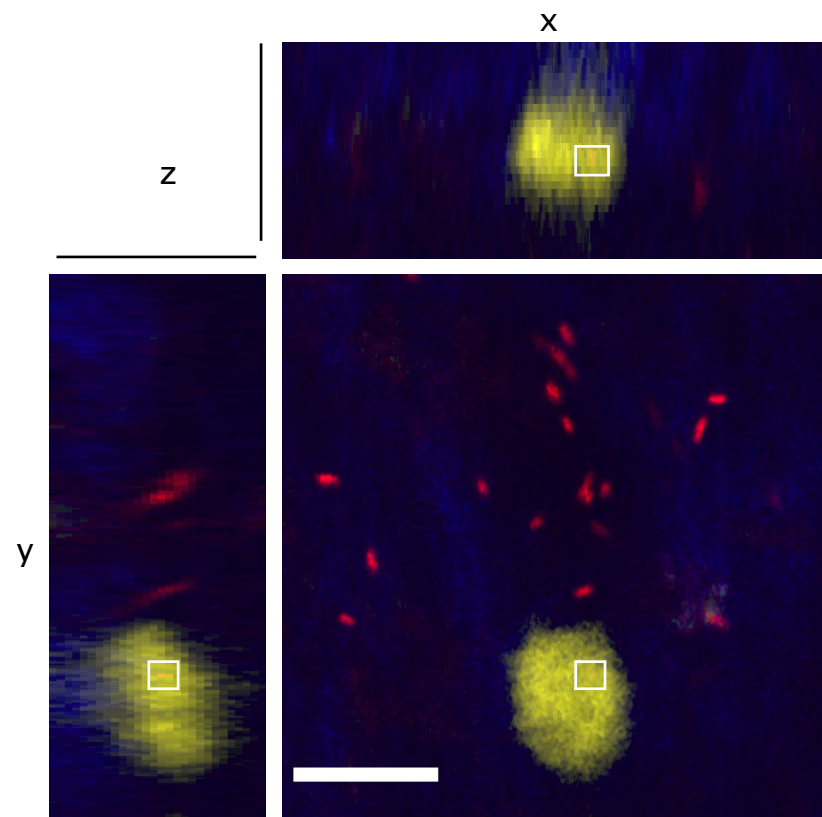

Supplement: Figure S4 — mCherry-BCG uptake by DDC. 2 h after intradermal mCherry:BCG (red) inoculation (2×10^5^ bacilli), a three-dimensional section of the ear was taken by 2P microscopy. A DDC (yellow) containing one intracellular BCG is visible (white box highlights BCG). Note that the DDC does not transform into a dendritic shaped cell. (0.38 MB PDF) [file ppat.1000222.s004.pdf]

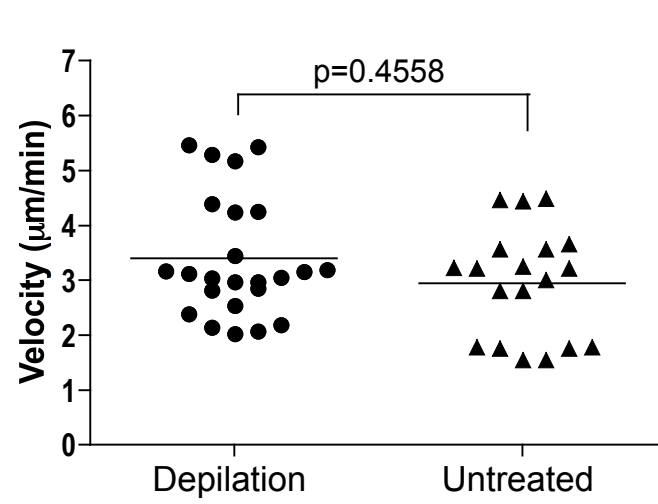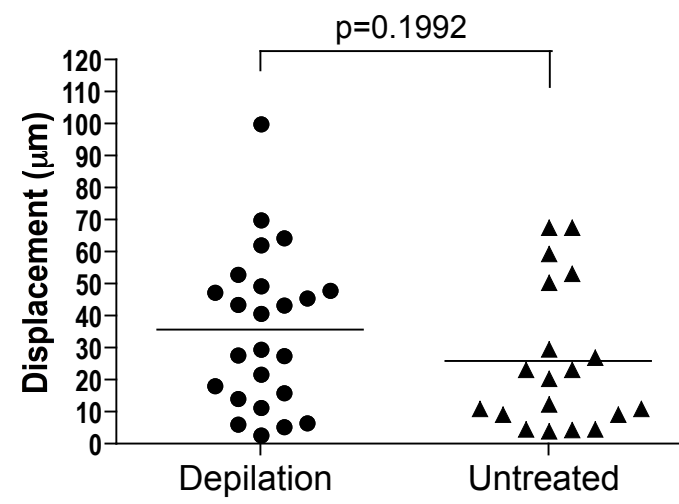

Supplement: Figure S5 — Hair removal does not influence the behavior of DDC in the skin. Experiments were performed with or without hair removal (n = 3 mice). (0.10 MB PDF) [file ppat.1000222.s005.pdf]
